# Potential Effect of DIMBOA (2,4-Dihydroxy-7-methoxy-1,4-benzoxazin-3-one) on Alleviating the Autotoxic Coumarin Stress in Alfalfa (*Medicago sativa*) Seedlings

**DOI:** 10.3390/life12122140

**Published:** 2022-12-19

**Authors:** Xiaolong Li, Shangli Shi, Xiaoyan Zhang, Changning Li, Huning Wang, Wenjuan Kang, Guoli Yin

**Affiliations:** 1 Key Laboratory of Grassland Ecosytem (Ministry of Education), College of Grassland Science, Gansu Agricultural University, Lanzhou 730070, China; 2 Centers for Grazing Land Ecosystem Sustainability, Gansu Agricultural University, Lanzhou 730070, China; 3 Pratacultural Engineering Laboratories of Gansu Province, Sino-U.S., Lanzhou 730070, China

**Keywords:** alfalfa, allelopathic, DIMBOA, coumarin, autotoxicity

## Abstract

The allelopathic theory has garnered considerable attention in the field of agricultural production for its efficient plant protection, rapid crop yield increase, and scientific establishment of the crop rotation system. To study the effects of the main maize allelochemical DIMBOA (2,4-Dihydroxy-7-methoxy-1,4-benzoxazin-3-one) on the growth and development of alfalfa under autotoxic coumarin stress, we treated alfalfa seedlings with DIMBOA under coumarin stress and non-stress conditions in this study. Results show that 0.0342 mM coumarin significantly inhibited alfalfa seed germination percentage(Gp), germination potential(GP), radicle length, germ length, seeding height, and simple viability index (SVI), with decreases of 37.29%, 59.91%, 7.60%, 30.90%, 13.27%, and 45.70%, respectively. An amount of 0.6 mM DIMBOA could promote alfalfa seed Gp, GP, radicle length, germ length, seeding height, dry fresh ratio, and SVI, with increases of 12.38%, 23.91%, 48.69%, 48.65%, 48.68%, 295.12%, and 67.17%, respectively. However, the addition of DIMBOA under conditions of coumarin stress could effectively alleviate coumarin effects on alfalfa seedlings. Coumarin + DIMBOA treatment for 24 h mainly decreased reactive oxygen species (ROSs) and malondialdehyde (MDA) as well as soluble protein and soluble sugar, increasing some antioxidant enzyme activities and antioxidant content to alleviate the oxidative damage of alfalfa caused by coumarin stress. Administration of treatment for 72 h significantly promoted the morphological development of alfalfa seeding roots. Administration of treatment for 96 h significantly enhanced the photosynthetic capacity of alfalfa seedlings. The results of principal component analysis demonstrated that chlorophyll b(Chl b)and net photosynthetic rate(*Pn*) were the key indicators for coumarin + DIMBOA treatment to promote photosynthesis in alfalfa seedlings. Additionally, root length, mean root diameter, and root volume were the key indicators of root growth and development. Coumarin + DIMBOA treatment primarily increased catalase(CAT), peroxidase (POD), and ascorbate peroxidase (APX) activity and antioxidants(ASA) while reducing MDA and superoxide anion radical(O_2_^•−^). This study strongly suggested that DIMBOA can effectively improve the tolerance of alfalfa seedlings to coumarin stress through a combination of effects on root morphology, photosynthesis, and physiological indicators.

## 1. Introduction

Alfalfa (*Medicago sativa*) is a high-yielding, high-protein, nutritious, and palatable perennial legume grass. It can effectively improve the yield and quality of meat, milk, and other livestock products, and plays an extremely important role in promoting the development process of grain-saving animal husbandry, with high economic and ecological value [1]. As a result, alfalfa has unique advantages in agricultural and livestock production and is widely grown globally [2]. In recent years, owing to the increasing demand for high-quality livestock products, the supply of alfalfa has reduced considerably, as it is a high-quality grass. This has resulted in an increased degree of intensive cultivation of alfalfa, and is subsequently compounded by the problem of continuous cropping obstacles due to autotoxicity, which have become one of the major problems limiting the sustainable development of the agriculture and livestock industry [3,4].

Alfalfa mainly secretes autotoxic substances in the soil via the root system, which directly inhibits the germination of secondary seeds and seedlings and produces indirect autotoxic effects by affecting soil physicochemical properties, affecting enzyme activity, and disrupting microbial balance [5]. Among them, coumarin is one of the important substances that produce autotoxic effects in alfalfa [6]. The study reported by Hedge and Miller demonstrated that the root length and branch number of alfalfa showed a curvilinear decrease with increasing concentration of coumarin [7]. The study of Chon et al. showed that coumarin is the most toxic and abundant autotoxic substance in alfalfa, and that 10^−3^ M of coumarin significantly inhibits root growth and interferes with the growth-division process of root epidermal cells in alfalfa [8]. Numerous studies have demonstrated that coumarins can negatively affect plants. For instance, Aliotta et al. found that coumarins can inhibit plant seed germination and seedling root growth [9]. Abenavoli et al. showed that coumarin can alter the size of wheat roots and thus reduce the uptake and utilization of nitrate nitrogen in wheat [10]. EL-Shahawy et al. [11] similarly concluded that coumarins have the ability to inhibit plant growth and development [11].

Establishing a rational farming system can effectively mitigate the effects of autotoxicity [12,13]. From the perspective of allelopathy theory, corn is the most suitable crop for rotation with alfalfa [14]. DIMBOA is the main resistance substance and allelochemical in maize [15]. Currently, studies on DIMBOA in maize have focused on its biosynthesis and resistance, and a large number of studies have reported that DIMBOA helps improve plant resistance to pests and diseases [16,17,18]. In addition, DIMBOA plays an extremely important role in allelopathy among plants and has an allelopathic potential for neighboring plants [19,20,21]. Perez found that there was a concentration-dependent effect of DIMBOA on the root system of cultivated oats, with DIMBOA promoting root growth when the concentration was <1.5 mM and inhibiting it when it was >1.5 mM [22]. Chen et al. showed that allelochemical DIMBOA can affect the community structure of soil microorganisms by altering the count of soil rhizosphere fungi in wheat, which in turn indirectly affects the growth and development of wheat [23].

In practical agricultural production, the problem of continuous cropping barriers, mainly autotoxicity, severely limits the yield and quality of alfalfa [3,4]. A large volume of studies have shown that establishing a rational cultivation system is an effective way to alleviate autotoxicity [12,13], and the study of Miller et al. has shown that maize is the most suitable crop for rotation with alfalfa from an allelopathic point of view [14]. However, little information is available on the effects of maize’s main allelochemical DIMBOA on the growth and development of alfalfa under coumarin stress. Therefore, in this study, we first treated alfalfa seeds with different concentrations of coumarin and DIMBOA to determine their effects on seed germination and sensitive concentrations. Then, we treated alfalfa seedlings with DIMBOA under coumarin stress and non-stress conditions and determined the morphological features, photosynthesis, and physiological indicators of alfalfa seedlings at different periods after treatment. The objectives of this study were (1) to determine whether DIMBOA treatment could alleviate the effects of coumarin stress on alfalfa seedlings, and (2) to determine the main factors of DIMBOA in alleviating growth inhibition of alfalfa seedlings under coumarin stress.

## 2. Materials and Methods

Based on the results of our previous screening tests on the autotoxicity effects of different varieties of alfalfa [24], the alfalfa variety 3105C, with its strong autotoxicity effect, was selected as the test material for this experiment.The test seeds were purchased from Beijing Zhengdao Seed Industry Co., Ltd.(Beijing, China).

### 2.1. Preparation of Coumarin and DIMBOA Treatment Solution

About 1.0 g of coumarin standard was accurately weighed and dissolved in 100 mL of distilled water. The volume was topped up to 1 L, and 1000 mg·L^−1^ of stock solution was prepared. The solution was stored in a 4 °C refrigerator. The stock solution was diluted to a concentration solution of 0.0034 (C1), 0.0342 (C2), and 0.3424 mM(C3). The DIMBOA standards were weighed and prepared into different concentrations of 0.15 (D1), 0.3 (D2), 0.45 (D3), 0.6 (D4), and 0.75 mM (D5) of DIMBOA treatment solutions. The coumarin and DIMBOA standards were purchased from SHANGHAI ZZBIO Co.,Ltd. Table 1 shows the specific information of standards.

### 2.2. Effects of Coumarin and DIMBOA Treatment on Seed Germination

Alfalfa 3105C seeds were treated with coumarin and DIMBOA treatment solutions, using the glass Petri dish method with distilled water as the control. The concentration gradient of coumarin treatment was CK (0 mM), C1 (0.0034 mM), C2 (0.0342 mM), and C3 (0.3424 mM), and the concentration gradient of DIMBOA treatment was CK (0 mM), D1 (0.15 mM), D2 (0.3 mM), D3 (0.45 mM), D4 (0.6 mM), and D5 (0.75 mM). Two layers of sterilized filter paper were placed at the bottom of a Petri dish with a diameter of 9 cm, and 30 alfalfa seeds were evenly placed in each Petri dish (sterilized with 3%NaOCl for 3 min, then soaked in 70% (*v*/*v*) ethanol for 1.5 min, and then washed with sterile distilled water four times). Four replicates were set up for each Petri dish. Different concentrations of 3.5 mL of coumarin and DIMBOA treatment solution were subsequently added, and an equal volume of distilled water was added to the control Petri dishes and placed in an artificial climate chamber (photoperiod 25 °C, 12 h; dark cycle 20 °C, 12 h) for germination. The treatment solution and distilled water were replenished with 1 mL every 2 days and were recorded on the seed germination table regularly every day until Day 7. On Day 7, germinated seed mass, radicle length, germ length, and germinated seed length were measured, and germination percentage (Gp), germination potential (GP), and simple viability index (SVI) were calculated according to Equation [25].

Gp = n_7_/n_total_ × 100% (1)

GP = n_3_/n_total_ × 100% (2)

SVI = Gp × Seeding height × 100(3)

In Formulas (1)–(3), n_7_ is the number of alfalfa seeds that germinated normally on Day 7, n_total_ is the total number of alfalfa seeds tested, and n_3_ is the number of alfalfa seeds that germinated normally on Day 3.

### 2.3. Effects of Coumarin and DIMBOA Treatment on Photosynthetic Physiology, Root Morphology, andAntioxidant Physiology of Seedlings

Alfalfa 3105C seedlings were cultivated using the sand culture method in plastic square pots of length × width × height (25 × 15 × 10 cm), with a 9 cm diameter nutrient bowl placed below the pot that was filled with quartz sand after sterilization via autoclaving (121 °C, 25 min). Alfalfa seeds that were saturated and uniformly sterilized using HgCl_2_ solution were selected and evenly sown in a nutrient bowl and placed in a light incubation room (14 h of light per day, light flux density of 400 μmol·m^−2^·s^−1^, day and night temperatures of 25 °C ± 1 °C and 20 °C ± 1 °C, respectively, and 60% relative humidity). After germination, 15 seedlings with uniform growth and distribution in each bowl were retained and watered with 300 mL of Hoagland nutrient solution every 2 days to ensure normal seedling growth [26]. At Day 45 after seedling emergence, 0.0342 mM coumarin (T1), 0.0342 mM coumarin + 0.6 mM DIMBOA (T2), and 0.6 mM DIMBOA (T3) treatment solutions were sprayed evenly in the nutrient pot, the control group was sprayed with an equal volume of distilled water, and three replications were set up. The photosynthetic parameters and root morphology of alfalfa seedlings were measured on days 1, 3, 5, 7, and 9 of treatment, and the roots of alfalfa seedlings were snap-frozen in liquid nitrogen and stored at −80 °C in an ultra-low temperature freezer for the determination of the physiological indicator.

### 2.4. Measurement Indicators and Methods

#### 2.4.1. Measurement of Photosynthetic Parameters

The GFS-3000 portable gas exchange and fluorescence system (Heinz-Walz, Effeltrich, Germany) was used to determine photosynthetic physiological indicators. The measurements were carried out between 9:00 and11:00 am with sufficient light, and the CO_2_ concentration and light intensity were 380 μmol·m^−2^·s^−1^ and 1200 μmol·m^−2^·s^−1^, respectively [27]. Net photosynthetic rate *(Pn*), transpiration rate *(E*), stomatal conductance *(gs*), and intercellular CO_2_ concentration *(Ci*) were calculated by referring to the method of von Caemmerer and Farquhar [28].

#### 2.4.2. Measurement of Chlorophyll

Chlorophyll (Chl a, Chl b, and carotenoid) was determined using the method reported by Wellburn [29].

#### 2.4.3. Measurement of Root Morphological Characteristics

The roots were rinsed with deionized water, and a bench scanner (model Epson Experssion 10000XL, EU-88, Seiko Epson Crop, Japan. Resolution: 300 dpi) was used to scan the root system. The steps were as follows: the rinsed roots were placed in a transparent tray, and distilled water was added to a depth of 10–15 mm to ensure complete dispersion of the root branches. The root system analysis system software WinRHIZO (Regent Instrument, Inc., Quebec, QC, Canada) was used to analyze the root images. The indicators measured were total root length, total root surface area, mean root diameter, root volume, and root tip count.

#### 2.4.4. Measurement of MDA and ROS (H_2_O_2_, OH^•^ and O_2_^•−^) Content

Malondialdehyde (MDA) was determined via the thiobarbituric acid method, following the method of Draper et al. [30]; hydrogen peroxide (H_2_O_2_) was determined through the KI colorimetric method, according to the method reported by Willekens et al. [31]; hydroxyl radical (OH^•^) concentration was determined using the 2-deoxy-D-ribose colorimetric method, following the method of Liu et al. [32]. Superoxide anion radical (O_2_^•−^) production rate was determined using the sulfanilic acid method, following the method of Elstner et al. [33].

#### 2.4.5. Measurement of Antioxidant Enzymes Activities and Antioxidant Content

We weighed 0.2 g of alfalfa seedling roots, and 4 mL of 50 mM phosphate buffer was added (PBS, containing 0.1 mmol·L^−1^ EDTA, 1% PVP, pH = 7.8). The roots were homogenized thoroughly in an ice bath, and the homogenate was centrifuged at 12000 r/min, for 20 min at 4 °C. The supernatant was collected, aliquoted, and stored in an ultra-low temperature freezer for analysis of enzyme activity [34]. Superoxide dismutase (SOD) activity was determined using the method of Giannopolitis et al. [35], peroxidase (POD) activity was determined using the method reported by Chance et al. [36], and catalase (CAT) activity was determined using the method of Havir et al. [37].

About 0.5 g of the test seedling roots were weighed and added into 5 mL of 50 mM PBS (containing 0.1 mM Na_2_EDTA, 0.3% Triton X-100, and 4% (*w*/*v*) PVP, pH = 7.5). They were homogenized thoroughly on ice, and the homogenate was centrifuged in a 16,000× *g* high-speed refrigerated centrifuge for 10 min at 4 °C. The supernatant was collected, aliquoted, and stored in an ultra-low temperature freezer to determine the antioxidant enzymes ascorbate peroxidase(APX) and glutathione reductase(GR) activities. APX and GR activities were determined using the method of Murshed et al. [38].

The levels of reduced ascorbic acid (AsA) and oxidized ascorbic acid (DHA) were determined by using the method reported by Murshed et al. [39]. We weighed 0.5 g of the alfalfa root sample and added 4 mL of 6% trichloroacetic acid (TCA, *w*/*v*). The sample was homogenized into a slurry on ice and centrifuged at 16,000× *g*, 4 °C for 10 min. The collected supernatant was used for the determination of AsA and total ascorbic acid. The DHA is the difference between total ascorbic acid and AsA.

Reduced glutathione (GSH) and oxidized glutathione (GSSG) were determined according to the method reported by Griffith [40]. We weighed 0.5 g of the test alfalfa root sample and added 4 mL of 7% (*w*/*v*) sulfosalicylic acid. The sample was homogenized thoroughly on ice, and the homogenate was centrifuged at 16,000 ×g, 4 °C for 10 min. The collected supernatant was used for the determination of total glutathione and GSSG. GSH is the difference between total glutathione and GSSG.

#### 2.4.6. Measurement of Soluble Protein and Soluble Sugar Content

Soluble sugar (SS) content was determined by using the anthrone colorimetric method [41]; soluble protein (SP) content was determined by using the Coomassie Brilliant Blue G-250 staining method [42].

## 3. Statistical Analysis

All data were analyzed using R software (version 4.0.2). A one-way ANOVA was used to compare the differences in photosynthetic indicators, root morphological indicators, antioxidant enzymes, antioxidants, SS, SP, ROS, and MDA between the treatment and control groups (significance level *p* < 0.05). The avo function in the agricolae package was used for the calculation. The duncan.test function in the agricolae package was used for multiple comparison analysis. The plyr package was used in technique for order of preference by similarity TOPSIS (Technique for Order of Preference by Similarity to Ideal Solution) analysis. Positive and negative contribution indicators and sensitive concentrations for both species were determined using the PCAtools package in R. The calculations were performed using the principal component analysis (PCA) function in the PCAtools package. The graphs in the paper were plotted using the ggplot2 and ggpubr packages in R software.

## 4. Results

### 4.1. Effects of DIMBOA and Coumarin on Alfalfa Seed Germination

**Figure 1:**
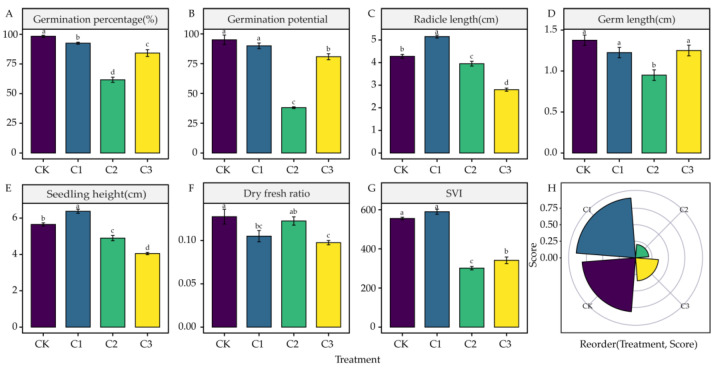
Effects of Coumarin Treatments on Alfalfa Seed Germination. (**A**) Germination percentage. (**B**) Germination potential. (**C**) Radicle length. (**D**) Germ length.(**E**)Seedling height. (**F**) Dry-to-fresh ratio. (**G**) SVI. (**H**) Comprehensive score ranking of the different treatments. Four colors represent the corresponding scores of CK, C1–C3 Treatments. C1: 0.0034 mM; C2: 0.0342 mM; C3: 0.3424 mM. The different lowercase letters indicate the significance in different treatments.

Compared to CK, a 0.0034 mM concentration of coumarin significantly reduced the germination and dry-to-fresh weight ratio of alfalfa seeds *(p* < 0.05) and significantly increased radicle length and seedling length *(p* < 0.05). At a 0.0342 mM concentration, all parameters were inhibited except for the dry-to-fresh weight ratio, which was significantly different from CK *(p* < 0.05). A 0.3424 mM concentration of coumarin had no significant effect on germ length; however, it significantly inhibited other parameters (Figure 1A–G). A comprehensive evaluation and analysis of several seed germination parameters (germination percentage, germination potential, radicle length, germ length, seedling height, dry-to-fresh weight ratio, and SVI) under coumarin concentration treatments(0 mM, 0.0034 mM, 0.0342 mM, 0.3424 mM) was performed using TOPSIS. According to the corresponding scores of the different treatments, the effect size of each treatment on alfalfa seed germination was ranked as 0.0034 mM>0 mM>0.3424 mM>0.0342 mM. This indicates that 0.0034 mM treatment promoted seed germination of alfalfa 3105C, whereas 0.0342 mM and 0.3424 mM had inhibitory effects. Among them, 0.0342 mM treatment had the strongest inhibitory effect (Figure 1H).

**Figure 2:**
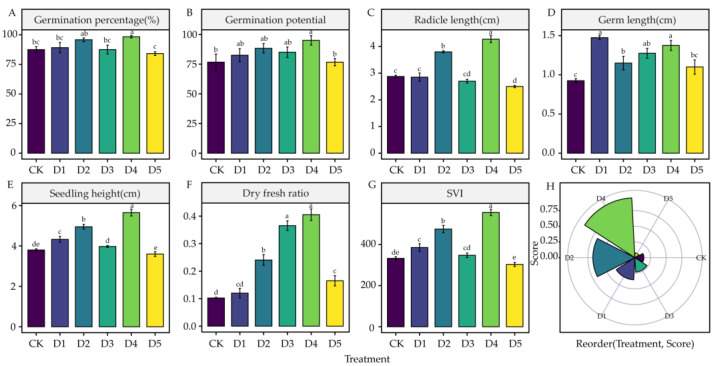
Effects of DIMBOA Treatments on Alfalfa Seed Germination. (**A**) Germination percentage. (**B**) Germination potential. (**C**) Radicle length. (**D**) Germ length. (**E**) Seedling height. (**F**) Dry-to-fresh ratio. (**G**) SVI. (**H**) Comprehensive score ranking of the different treatments. Six colors represent treatments of CK, D1–D5 Treatments. D1: 0.15 mM; D2: 0.3 mM; D3: 0.45 mM; D4: 0.6 mM; D5: 0.75 mM. The different lowercase letters indicate the significance in different treatments.

DIMBOA treatment at a 0.6 mM concentration significantly increased the germination rate and germination potential of alfalfa seeds compared to CK *(p* < 0.05) (Figure 2A,B). Both 0.3 mM and 0.6 mM treatments significantly increased the radicle length of alfalfa seeds compared to those of CK *(p* < 0.05), while 0.75 mM treatment significantly inhibited radicle growth *(p* < 0.05) (Figure 2C). Germ length was significantly increased *(p* < 0.05) in 0.15 mM, 0.3 mM, 0.45 mM, and 0.6 mM treatments compared to that in CK (Figure 2D). Meanwhile, 0.15 mM, 0.3 mM, and 0.6 mM significantly increased the seedling length of alfalfa seeds *(p* < 0.05) (Figure 2E). The dry-to-fresh weight ratio increased significantly *(p* < 0.05) under 0.3 mM, 0.45 mM, 0.6 mM, and 0.75 mM treatments compared with that in CK, with 0.6 mM showing the greatest increase (Figure 2F). Compared with CK, we observed that 0.15 mM, 0.3 mM, and 0.6 mM significantly increased SVI *(p* < 0.05) (Figure 2G). Comprehensive TOPSIS analysis showed that the magnitude of the promoting effect of each treatment on alfalfa seed germination was ranked in the following order: 0.6 mM > 0.3 mM > 0.15 mM > 0.45 mM > CK > 0.75 mM. This indicates that the 0.6 mM, 0.3 mM, 0.15 mM, and 0.45 mM treatments promoted seed germination of alfalfa 3105C, whereas 0.75 mM inhibited it, with 0.6 mM treatment demonstrating the greatest promoting effect (Figure 2H).

### 4.2. Effects of DIMBOA and Coumarin on Photosynthetic Parameter Activities of Alfalfa

**Figure 3:**
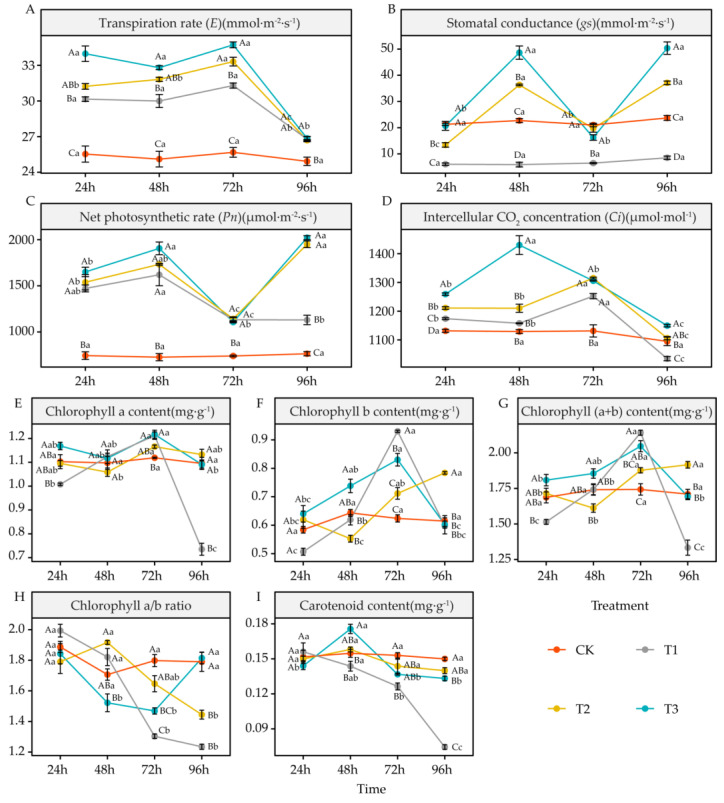
Effects of DIMBOA and Coumarin Treatments on Photosynthetic Parameter Activities of Alfalfa. (**A**) *E*; (**B**) *gs*; (**C**) *Pn*; (**D**) *Ci*; (**E**) Chlorophyll a content; (**F**) Chlorophyll b content; (**G**) Chlorophyll (a + b) content; (**H**) Chlorophyll (a/b) ratio; (**I**) Carotenoid content. Four colors represent treatments of CK, T1, T2, T3 treatments. T1: 0.0342 mM Coumarin; T2: 0.0342 mM Coumarin + 0.6 mM DIMBOA; T3: 0.6 mM DIMBOA. The uppercase letters indicate significant differences between different treatments at the same time. The lowercase letters indicate significant differences between different times under the same treatment. The same below.

Different treatments and different treatment durations produced different degrees of effects on gaseous exchange parameters in alfalfa 3105C, with coumarin (T1), coumarin + DIMBOA (T2), and DIMBOA treatments (T3) significantly enhancing transpiration rate *(E*) in alfalfa *(p* < 0.05). Coumarin + DIMBOA (T2) and DIMBOA treatments (T3) were more effective than coumarin (T1) treatment (Figure 3A). Furthermore, coumarin (T1) treatment significantly reduced stomatal conductance *(gs*) in alfalfa *(p* < 0.05), whereas coumarin + DIMBOA (T2) and DIMBOA treatment (T3) significantly increased stomatal conductance *(p* < 0.05) at 48 h and 96 h, respectively (Figure 3B). All treatments significantly increased the net photosynthetic rate *(Pn*) of alfalfa (*p* < 0.05), with coumarin + DIMBOA (T2) and DIMBOA treatment (T3) showing significantly greater promotion than coumarin (T1) treatment *(p <* 0.05) at 96 h (Figure 3C). In addition, coumarin + DIMBOA (T2) and DIMBOA treatment (T3) also had a greater elevation effect on intercellular CO_2_ concentration (*Ci*) than coumarin (T1) treatment, and at 96 h, the intercellular CO_2_ concentration of coumarin (T1) treatment was significantly lower than even that of CK *(p* < 0.05) (Figure 3D).

Different treatments and different treatment durations also affected the photosynthetic pigment level of alfalfa 3105C to varying degrees; the Chl a of coumarin treatment (T1) was significantly lower than that of CK at 24 h and 96 h (*p* < 0.05), while the Chl a of coumarin + DIMBOA (T2) and DIMBOA treatment (T3) was not significantly different compared to that of CK (Figure 3E). The Chl b of coumarin treatment (T1) was significantly lower than that of CK at 24 h (*p* < 0.05), then gradually increased to be significantly larger than CK at 72 h (*p*< 0.05), but decreased to CK level again at 96 h. Coumarin + DIMBOA (T2) treatment increased Chl b at 96 h, which was significantly different compared to that of CK (*p* < 0.05). The Chl b of DIMBOA treatment (T3) was significantly greater than that of CK (*p* < 0.05) only at 72 h (Figure 3F); the trend of Chl (a + b) under the three treatments was identical to that of Chl b. The difference was that the Chl (a + b) of coumarin treatment (T1) was significantly lower than that of CK (*p* < 0.05) at 96 h, and the Ch1 (a + b) of coumarin + DIMBOA (T2) treatment was significantly greater than that of CK (*p* < 0.05) (Figure 3G). Compared to CK, Chl (a/b) of coumarin treatment (T1) was significantly decreased at 72 h and 96 h (*p* < 0.05). In contrast, coumarin + DIMBOA (T2) treatment was not significantly different compared to CK between 24 and72 h and only decreased at 96 h (*p* < 0.05). Chl (a/b) gradually decreased after DIMBOA treatment (T3) and was significantly lower than that of CK at 72 h (*p <* 0.05). It then gradually increased and was not significantly different from CK at 96 h (Figure 3H). Carotenoid gradually decreased after coumarin treatment (T1) and was significantly lower than that of CK at 72 h and 96 h (*p* < 0.05), whereas the carotenoid in coumarin + DIMBOA (T2) treatment was not significantly different from that in CK. DIMBOA treatment (T3) significantly increased carotenoid at 48 h (*p* < 0.05) (Figure 3I).

### 4.3. Effects of DIMBOA and Coumarin on Root Morphological Characteristics of Alfalfa

**Figure 4:**
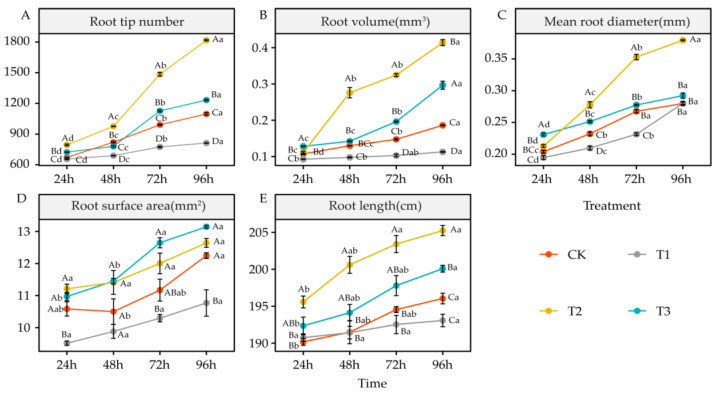
Effects of DIMBOA and Coumarin Treatments on Root Characteristics of Alfalfa. (**A**) Root tip number; (**B**) Root volume; (**C**) Mean root diameter; (**D**) Root surface area; (**E**) Root length. Four colors represent treatments of CK, T1, T2, T3 Treatments. T1: 0.0342 mM Coumarin; T2: 0.0342 mM Coumarin + 0.6 mM DIMBOA; T3: 0.6 mM DIMBOA.

The root tip count of alfalfa seedlings in each treatment group showed an increasing trend as treatment duration increased. Among them, the root tip count after 48 h of coumarin treatment (T1) (*p* < 0.05) was significantly lower than that of CK. However, the root tip count under coumarin + DIMBOA treatment (T2) was significantly greater than that of CK (*p* < 0.05). DIMBOA treatment (T1) also significantly increased the root tip count of alfalfa seedlings at 24 h, 72 h, and 96 h (*p* < 0.05) (Figure 4A). The trend of root volume of alfalfa seedlings under each treatment was basically the same as that of root tip count. Similarly, coumarin treatment (T1) significantly inhibited root volume growth (*p* < 0.05), while both coumarin + DIMBOA (T2) treatment and DIMBOA alone (T3) significantly promoted root volume growth (*p* < 0.05) (Figure 4B). The mean root diameter of alfalfa under coumarin treatment (T1) at 48 h and 72 h was significantly lower than that of CK (*p* < 0.05), while coumarin + DIMBOA treatment (T2) significantly increased the mean root diameter after 48 h (*p* < 0.05). DIMBOA-treated plants (T3) had a significantly larger mean root diameter than those of CK at 24 h and 48 h (*p* < 0.05); however, its value was essentially the same as CK after 72 h (Figure 4C). Coumarin treatment (T1) significantly inhibited the increase in alfalfa root surface area at 24 h and 96 h compared to CK (*p* < 0.05), while coumarin + DIMBOA treatment (T2) and DIMBOA alone (T3) had a slight contribution to the increase in root surface area, with the differences being non-significant (Figure 4D). The root length of alfalfa under coumarin treatment (T1) was not significantly different to that of CK, whereas coumarin + DIMBOA treatment (T2) significantly increased the root length of alfalfa (*p* < 0.05). DIMBOA treatment (T3) had a slight promotion effect on the root length of alfalfa, but the difference was not significant (Figure 4E).

### 4.4. Effects of DIMBOA and Coumarin on ROS and Malondialdehyde of Alfalfa

**Figure 5:**
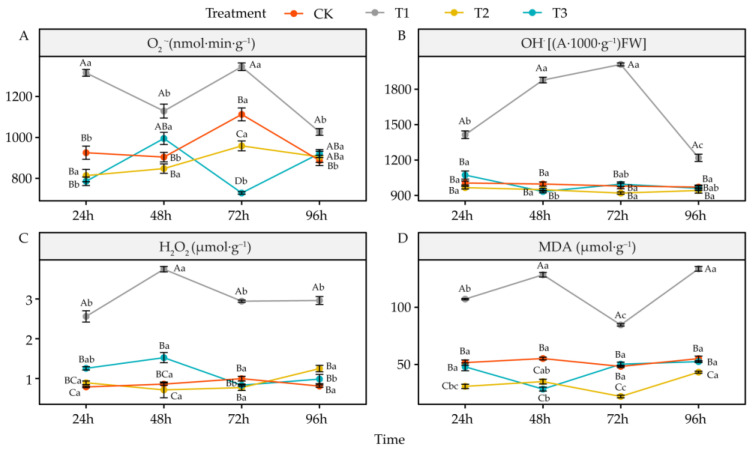
Effects of DIMBOA and Coumarin Treatments on ROS Production and Peroxidation Activities of Alfalfa. (**A**) O_2_^•−^ content; (**B**) OH^•^ content; (**C**) H_2_O_2_ content; (**D**) MDA content. Four colors represent treatments of CK, T1, T2, T3 Treatments. T1: 0.0342 mM Coumarin; T2: 0.0342 mM Coumarin + 0.6 mM DIMBOA; T3: 0.6 mM DIMBOA.

Compared to CK, coumarin treatment (T1) significantly increased the content of O_2_^•−^, OH^•^, H_2_O_2_, and MDA in the root system of alfalfa seedlings at all the time points (*p* < 0.05) (Figure 5A–D). Coumarin + DIMBOA treatment (T2) significantly reduced O_2_^•−^ at 72 h (*p* < 0.05), while OH^•^ and H_2_O_2_ were not significantly different from CK, and MDA was significantly reduced at all the time nodes *(p* < 0.05) (Figure 5D). DIMBOA treatment (T3) significantly reduced O_2_^•−^ at 72 h (*p* < 0.05) (Figure 5A) and MDA at 48 h (*p* < 0.05) (Figure 5D). OH^•^ and H_2_O_2_ remained at the same levels as those of CK (Figure 5B,C).

### 4.5. Effects of DIMBOA and Coumarin on Antioxidant Enzymes Activities of Alfalfa

**Figure 6:**
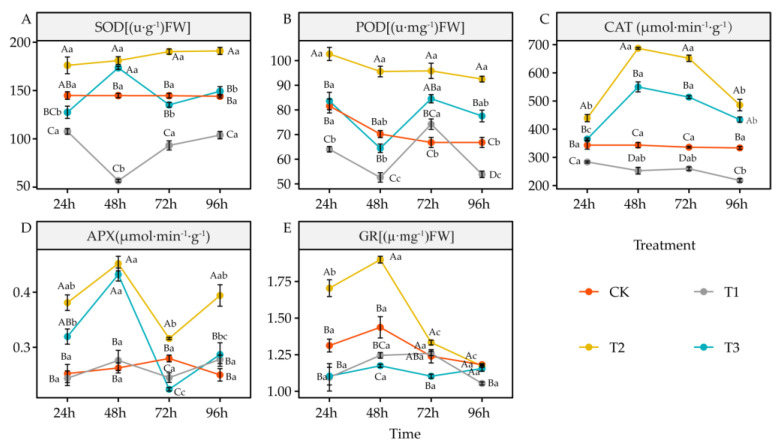
Effects of DIMBOA and Coumarin treatments on Antioxidant Enzymes Activities of Alfalfa Seedlings. (**A**) SOD activity; (**B**) POD activity; (**C**) CAT activity; (**D**) APX activity; (**E**) GR activity. Four colors represent treatments of CK, T1, T2, T3 Treatments. T1: 0.0342 mM Coumarin; T2: 0.0342 mM Coumarin + 0.6 mM DIMBOA; T3: 0.6 mM DIMBOA.

Compared to CK, coumarin treatment (T1) significantly reduced SOD activity (*p <* 0.05). POD activity was significantly inhibited at 24 h, 48 h, and 96 h (*p* < 0.05), CAT activity was significantly reduced at all the time points (*p* < 0.05), APX activity was significantly reduced at 72 h (*p* < 0.05), and GR activity was significantly inhibited at 24 h, 48 h, and 96 h (*p* < 0.05) (Figure 6A–E). Coumarin + DIMBOA treatment (T2) significantly increased SOD activity at 48 h, 72 h, and 96 h (*p* < 0.05), significantly increased POD, CAT, and APX activities at all time points *(p* < 0.05), and significantly increased GR activity at 24 h and 48 h *(p* < 0.05) (Figure 6A–E). DIMBOA treatment (T3) significantly increased SOD activity at 48 h (*p* < 0.05), significantly increased POD activity at 72 h and 96 h (*p* < 0.05), significantly increased CAT activity at 48 h, 72 h, and 96 h (*p* < 0.05), and significantly increased APX activity at 48 h (*p* < 0.05). However, it significantly decreased APX activity at 72 h (*p* < 0.05) and significantly decreased GR activity at 48 h and 72 h (*p* < 0.05) (Figure 6A–E).

### 4.6. Effects of DIMBOA and Coumarin on Antioxidants of Alfalfa

**Figure 7:**
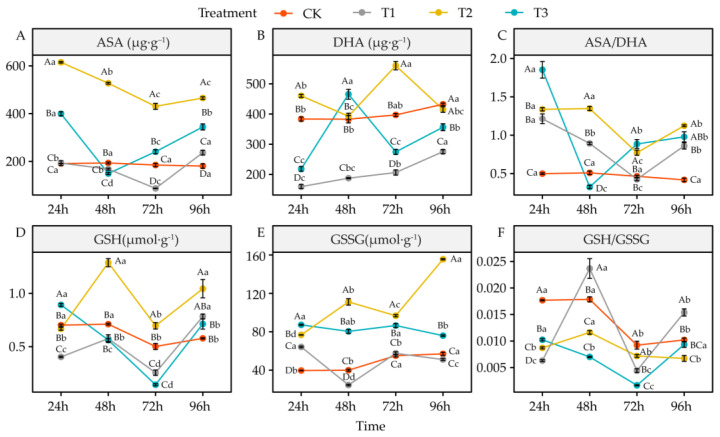
Effects of DIMBOA and Coumarin Treatments on Antioxidants of Alfalfa Seedlings. (**A**) ASA content; (**B**) DHA content; (**C**) ASA/DHA content; (**D**) GSH content; (**E**) GSSG content; (**F**) GSH/GSSG ratio. Four colors represent treatments of CK, T1, T2, T3 Treatments. T1: 0.0342 mMCoumarin; T2: 0.0342 mM Coumarin + 0.6 mM DIMBOA; T3: 0.6 mM DIMBOA.

Compared to CK, coumarin treatment (T1) significantly reduced ASA in alfalfa seedling roots at 48 h and 72 h (*p* < 0.05), while it significantly increased it at 96 h (*p* < 0.05). It significantly reduced DHA (*p* < 0.05), significantly increased ASA/DHA ratio at 24 h, 48 h, and 96 h (*p* < 0.05), significantly decreased GSH (*p* < 0.05) at 24 h and 72 h, significantly increased GSSG (*p* < 0.05) at 24 h yet significantly decreased it (*p* < 0.05) at 48 h, and significantly decreased GSH/GSSG ratio (*p* < 0.05) at 24 h and 72 h, yet significantly increased it again at 48 h and 96 h (*p* < 0.05) (Figure 7A–F). Compared to CK, coumarin + DIMBOA treatment (T2) significantly increased ASA, ASA/DHA ratio, and GSSG (*p* < 0.05), significantly increased DHA at 24 h and 72 h (*p* < 0.05), significantly increased GSH at 48 h, 72 h, and 96 h (*p* < 0.05), and significantly decreased GSH/GSSG ratio (*p* < 0.05) at 24 h, 48 h, and 96 h (Figure 7A–F). Compared to CK, DIMBOA treatment (T3) showed a significant increase in ASA at 24 h, 72 h, and 96 h (*p* < 0.05) and a significant decrease at 48 h (*p* < 0.05). DHA was significantly decreased at 24 h, 72 h, and 96 h (*p* < 0.05), while it was significantly increased at 48 h (*p* < 0.05). The ASA/DHA ratio was increased significantly at 24 h, 72 h, and 96 h, while it was decreased significantly at 48 h (*p* < 0.05), and the GSH was increased significantly at 24 h but decreased significantly at 72 h (*p* < 0.05). GSSG was increased significantly *(p* < 0.05), and GSH/GSSG ratio was decreased significantly (*p* < 0.05) at 24 h, 48 h, and 72 h (Figure 7A–F).

### 4.7. Effects of DIMBOA and Coumarin on Soluble Protein and Soluble Sugar Content of Alfalfa

**Figure 8:**
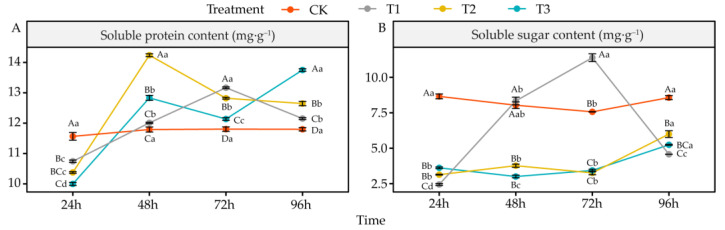
Effects of DIMBOA and Coumarin Treatments on Soluble Protein and Soluble Sugar Content of Alfalfa. (**A**) Soluble protein content; (**B**) Soluble sugar content. Four colors represent treatments of CK, T1, T2, T3 Treatments. T1: 0.0342 mM Coumarin; T2: 0.0342 mM Coumarin + 0.6 mM DIMBOA; T3: 0.6 mM DIMBOA.

Compared to CK, coumarin treatment (T1) significantly reduced soluble protein in alfalfa seedling roots at 24 h (*p* < 0.05) and significantly increased it at 72 h and 96 h (*p* < 0.05). The soluble sugar was decreased significantly at 24 h and 96 h and increased significantly (*p* < 0.05) at 72 h (Figure 8A,B). Compared to CK, coumarin + DIMBOA treatment (T2) significantly reduced soluble protein at 24 h and significantly increased it at 48 h, 72 h, and 96 h (*p* < 0.05), as well as significantly reducing soluble sugar at all time points (*p* < 0.05) (Figure 8A,B). Compared to CK, DIMBOA treatment (T2) significantly decreased soluble protein at 24 h and significantly increased it at 48 h, 72 h, and 96 h (*p* < 0.05), as well as significantly decreasing soluble sugar (*p* < 0.05) (Figure 8A,B).

### 4.8. Effects of DIMBOA and Coumarin on Photosynthetic, Root Characteristics, and Physiological Indicators of Stress Resistance of Alfalfa

**Figure 9:**
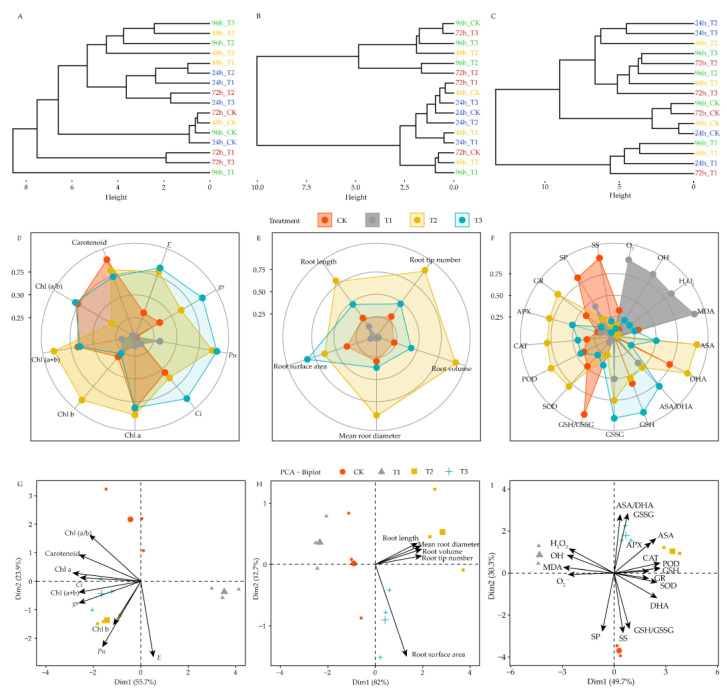
Photosynthetic indicators, root characteristics, and physiological indicators of stress resistance cluster analysis diagram and radar chart. (**A**–**C**) represents the different treatments cluster analysis diagram; (**D**–**F**) represents the different treatments radar chart; (**G**–**I**) represents the different treatments PCA-Biplot. Four colors represent treatments of CK, T1, T2, T3 Treatments.

Cluster analysis was used to comprehensively evaluate and analyze multiple photosynthesis-related parameters, multiple root morphological features, and multiple physiological indicators under different treatments and different treatment durations. Based on the distances between the different treatments, it was evident that the photosynthetic parameters, root morphological features, and physiological indicators of each treatment showed the greatest differences from CK at 96 h, 72 h, and 24 h, respectively (Figure 9A–C). Therefore, in this study, 96 h, 72 h, and 24 h were used as sensitive times to affect photosynthesis, root morphological features, and physiological processes, respectively.

Radar charts showed that coumarin treatment (T1) significantly reduced Chl a, Chl (a + b), carotenoid, and Chl (a/b) ratio of alfalfa seedlings at 96 h compared to CK, while it significantly reduced transpiration rate (*E*), stomatal conductance (*gs*), and intercellular CO_2_ concentration (*Ci*). Compared to CK, coumarin + DIMBOA treatment (T2) significantly increased Chl b, Chl (a/b) ratio, transpiration rate (*E*), stomatal conductance (*gs*), and net photosynthetic rate (*Pn*), but significantly decreased Chl (a + b). DIMBOA treatment (T3) significantly increased transpiration rate (*E*), stomatal conductance (*gs*), net photosynthetic rate (*Pn*), and intercellular CO_2_ concentration (*Ci*) (Figure 9D). At 72 h, in terms of root morphological features, coumarin treatment (T1) significantly reduced root tip count, root volume, root mean diameter, root surface area, and root length compared to CK. Both coumarin + DIMBOA treatment (T2) and DIMBOA treatment (T3) significantly increased the morphological features of each root system, but coumarin + DIMBOA treatment (T2) had a greater promotion effect (Figure 9E). At 24 h, in terms of physiological parameters, coumarin treatment (T1), compared to CK, significantly increased O_2_^•−^, OH^•^, H_2_O_2_, and MDA. It decreased SOD, POD, CAT, and GR activities, as well as soluble protein, soluble sugar, and DHA, GSH and GSH/GSSG ratio. Compared to CK, coumarin + DIMBOA treatment (T2) significantly decreased O_2_^•−^, OH^•^, H_2_O_2_, and MDA, as well as soluble protein and soluble sugar; increased SOD, POD, CAT, APX, and GR activities; and increased ASA, DHA, and GSSG and ASA/DHA ratio. DIMBOA treatment (T3) significantly increased APX activity and decreased SOD and GR activity, whereas it decreased soluble protein and soluble sugar content. It increased the ASA, GSH and GSSG and decreased the DHA and GSH/GSSG ratio (Figure 9F).

Based on the results of the above analysis, PCA analysis was used to determine the key parameters of each indicator at the respective sensitive times of photosynthesis-related indicators at 96 h, root morphology at 72 h, and physiological indicators at 24 h. The results show that CK, coumarin treatment (T1), coumarin + DIMBOA treatment (T2), and DIMBOA treatment (T3) were divided into four groups based on Dim1 and Dim2 at 96 h. The contribution of Dim1 and Dim2 to the included information was approximately 79.6% (55.7% + 23.9%). The distance and angle between the geometric center of each treatment and each indicator showed that at 96 h, coumarin + DIMBOA treatment (T2) increased Chl b and net photosynthetic rate (*Pn*), while DIMBOA treatment (T3) increased Chl (a + b) and stomatal conductance (*gs*). Various photosynthetic indicators showed low or negative correlations with CK and coumarin (T1) treatments (Figure 9G). In terms of root morphology, the treatments were divided into four groups by Dim1 and Dim2 at 72 h. The contribution of Dim1 and Dim2 to the information contained was approximately 94.7% (82% + 12.7%). Coumarin + DIMBOA treatment (T2) increased root length, mean root diameter, and root volume, whereas DIMBOA treatment (T3) only increased the root surface area. In contrast, various root morphological feature parameters showed low or negative correlations with CK and coumarin treatment (T1) (Figure 9H). In terms of physiological parameters, the treatments were divided into four groups according to Dim1 and Dim2 values at 24 h. The contribution of Dim1 and Dim2 to the included information was approximately 76.6% (47.6% + 29%). The distance and angle between the geometric center of each treatment and each indicator showed that coumarin + DIMBOA treatment (T2) increased ASA and CAT, POD, and APX activity, but decreased MDA and O_2_^•−^. DIMBOA treatment (T3) increased GSSG and ASA/DHA ratio, but decreased SP and GSH/GSSG ratio. Coumarin treatment (T1) increased H_2_O_2_, O_2_^•−^, OH^•^, and MDA, but decreased SOD, GR activity, GSH, and DHA (Figure 9I).

## 5. Discussion

### 5.1. Effects of DIMBOA and Coumarin on Alfalfa Seed Germination

Autotoxicity is one of the main reasons limiting the production and sustainable use of alfalfa [43]. Coumarin is the most abundant and toxic autotoxic substance in alfalfa [8]. Hall et al. found that autotoxic substances can substantially reduce seed germination [44]. Furthermore, autotoxic substances can affect the growth and development of the seed radicle and germ, thereby inhibiting seed germination [45,46,47]. In this study, we found a low-promoting and high-suppressing concentration effect of coumarin on the germination of alfalfa 3105C seeds. For C1 (0.0034 mM), the treatment of coumarin significantly increased radicle length and seedling length. The combined TOPSIS analysis also showed that the C1 (0.0034 mM) treatment scored higher than CK, showing a facilitative effect. Almost all seed germination parameters were inhibited under C2 (0.0342 mM) concentration of coumarin treatment (Figure 1A–G), and TOPSIS combined analysis also showed that C2 (0.0342 mM) treatment scored the lowest. Therefore, 0.0342 mM was a sensitive concentration for the autotoxic effect of alfalfa 3105C in response to coumarin. Abdulrahman et al. showed that different concentrations of autotoxic substances have different effects on the germination of recipient plants, which coincides with the results of the present study [5]. High concentrations of autotoxic substances inhibit seed germination for a variety of reasons. They can affect key enzymes required for seed germination, including phosphatase and cellulase, as well as inhibit cell division, resulting in a lack of essential energy and intermediates required for metabolism, which consequently reduces seed viability and inhibits germination [48,49].

DIMBOA is a broad-spectrum resistant substance in maize and other grasses. Numerous studies have reported that DIMBOA can enhance the resistance of plants to pests and diseases and promote healthy plant growth. A previous study found that DIMBOA is a major factor in resistance of leaves to Asian corn borer [50]. Additionally, a certain concentration of DIMBOA has an allelopathic promotion effect on plants [20,21]. In this study, according to the comprehensive TOPSIS analysis, the magnitude of the effects of different concentrations of DIMBOA on seed germination was ranked as D4 (0.6 mM) > D2 (0.3 mM) > D1 (0.15 mM) > D3 (0.45 mM) > CK (0 mM) > D5 (0.75 mM), where the same concentration effect of promotion at low concentrations and inhibition at high concentrations was observed. Among them, D4 (0.6 mM) treatment significantly promoted various germination parameters and had the best promotion effect. In contrast, D5 (0.75 mM) treatment exhibited a negative effect by inhibiting radicle growth. We believe that the main reason for the concentration effect is that DIMBOA is a biologically active substance, and Calabrese et al. demonstrated that biologically active substances can exhibit completely opposite effects at high and low concentrations [51]. In addition, this study found that 0.6 mM was the most sensitive concentration for DIMBOA to promote the germination of alfalfa 3105C seeds, where the promotion effect was the most significant.

### 5.2. Effects of DIMBOA and Coumarin on Photosynthetic Parameters of Alfalfa

Photosynthesis is an important pathway for carbon fixation and dry matter accumulation in plants. Previous studies have shown that autotoxic substances can reduce the photosynthetic capacity of plants by disrupting cell membrane integrity, affecting stomatal respiration intensity, and accelerating chlorophyll decomposition [52], as well as inhibiting the formation of photosynthetic products and reducing plant yield [53,54]. In this study, we found that coumarin reduced the photosynthetic efficiency of alfalfa by inhibiting stomatal conductance and intercellular CO_2_ concentration, whereas DIMBOA promoted photosynthesis by enhancing various gas exchange parameters. In addition, the addition of DIMBOA under coumarin treatment could effectively improve the inhibition effect of coumarin on photosynthesis in alfalfa by enhancing stomatal conductance, net photosynthetic rate, and intercellular CO_2_ concentration. Studies have shown that the main reasons for the inhibition of photosynthesis in plants are stomatal and non-stomatal factors [55]. Hao et al. found that stomatal limitation was characterized by a simultaneous decrease in stomatal conductance and intercellular CO_2_ concentration, whereas non-stomatal limitation was characterized by a decrease in stomatal conductance and a constant or even an increase in intercellular CO_2_ concentration [56]. This suggests that non-stomatal factors are the main cause of inhibition of photosynthetic efficiency in alfalfa seedlings by autotoxicity. In contrast, DIMBOA can ameliorate photosynthetic inhibition induced by autotoxic stress through the regulation of non-stomatal factors.

Chlorophyll is the main pigment for photosynthesis in plants, and its level is one of the important factors affecting the intensity of photosynthesis [57]. Eunbhlling et al. found that allelochemicals can reduce the chlorophyll level in leaves and thus affect normal photosynthesis in plants [58]. Cheng et al. showed that allelochemicals cause a decrease in photosynthetic efficiency of plants by promoting the breakdown and inhibiting the synthesis of photosynthetic pigments [59]. In the present study, coumarin treatment significantly reduced Chl a, Chl (a + b), Chl (a/b), and carotenoids at 96 h. DIMBOA treatment alone could maintain various photosynthetic pigments in alfalfa at essentially the same level as CK. The addition of DIMBOA under coumarin treatment not only maintained the Chl a and carotenoid at the same level as CK, but also increased the Chl b and Chl (a + b) at 96 h. This indicated that coumarin treatment significantly inhibited photosynthesis in alfalfa, while DIMBOA had fewer effects on its photosynthesis. When the two chemicals were used to treat alfalfa, they not only ameliorated the negative effects produced by coumarins, but also exerted some promotional effects by increasing the levels of Chl b and Chl (a + b). Interestingly, this study found that the addition of DIMBOA under coumarin treatment had a stronger promotion effect than DIMBOA alone. Studies have shown that the allelopathy effect of multiple allelopathic substances is significantly better than that of a single allelopathic substance [60,61]. We speculate that it may be attributable to the interaction between two allelochemicals, coumarin and DIMBOA, in which coumarin stimulates DIMBOA activity to increase, which in turn produces a greater effect.

### 5.3. Effects of DIMBOA and Coumarin on Root Characteristics of Alfalfa

Plants mainly rely on the root system to absorb and utilize water and nutrients in the soil, and the growth and development of the root system complements the growth of the aboveground organs of the plant, whereas the root morphological structure is the main indicator directly reflecting the growth and development of the plant root system [62]. Vaughan et al. found that autotoxic substances inhibited both the primary and secondary roots of plants [63]. Chon et al. showed that coumarin, an autotoxic substance of alfalfa, significantly inhibited elongation growth and cell division in root tip cells [8]. In this study, the root tip count, root volume, mean root diameter, and root surface area of alfalfa seedlings under coumarin treatment were significantly lower than those of the control, which indicated that coumarin could inhibit the root growth and development of alfalfa seedlings, in agreement with the findings of Chon et al. Perez found that certain concentrations of DIMBOA can promote oat root growth [22]. In this study, the root tip count and root volume of alfalfa seedlings under DIMBOA treatment increased significantly, indicating that DIMBOA had a stimulating effect on the root growth of alfalfa seedlings. In addition, the addition of DIMBOA under coumarin treatment improved the inhibitory effect of autotoxic substances on alfalfa seedling roots by increasing root tip number, root volume, mean root diameter, and root length. Similar to the effect on chlorophyll, the addition of DIMBOA under coumarin treatment had a stronger promotion effect on alfalfa seedling root growth than DIMBOA alone.

### 5.4. Alteration of Oxidative Damage in Alfalfa under DIMBOA and Coumarin

Accumulation of reactive oxygen species (ROS) and membrane lipid peroxidation are the main physiological features of plant response to stress [64]. Plants subjected to autotoxicity produce large amounts of ROS, such as O_2_^•−^, H_2_O_2_, and OH^•−^, and the accumulation of large amounts of ROS can lead to cell membrane damage [65]. At the same time, the accumulation of ROS can lead to peroxidation or delipidation of unsaturated fatty acids in cell membranes, which are degraded to small molecules MDA step by step [66]. When ROS accumulate in plants, oxidative damage can be counteracted by increasing the level of non-enzymatic antioxidants and the activity of antioxidant enzymes [67]. SOD, POD, CAT, APX, and GR are important antioxidant enzymes in plants, and the AsA–GSH cycle is an important non-enzymatic antioxidant system. Both pathways can directly scavenge ROS from plants under stress [68]. Osmoregulation of plant cells is one of the important ways in which plants adapt to their environment and resist stress [69]. The accumulation of osmoregulatory substances has also been found to be an indicator of damage to plant tissues [70]. In this study, 0.0342 mM coumarin treatment resulted in a large accumulation of ROS in the root cells of alfalfa seedlings while concurrently inhibiting most of the antioxidant enzyme activities, and the levels of antioxidants as well as osmoregulatory substances were reduced. This led to a decrease in the ability of alfalfa root system to scavenge ROS and intracellular redox imbalance, which caused oxidative damage to the root system of alfalfa seedlings. Batish and Hong et al. found that phenolic acid autotoxic substances cause damage to plants mainly by disrupting redox balance, which is consistent with the results of the present study [71,72]. When 0.0342 mM coumarin and 0.6 mM DIMBOA were used to treat alfalfa seedlings together, they did not cause ROS accumulation and even reduced O_2_^•−^ and MDA, thereby decreasing the degree of membrane lipid peroxidation while promoting the activity of some antioxidant enzymes and increasing the level of some antioxidants and osmoregulatory substances. This consequently increased the resistance of the alfalfa seedlings to autotoxic coumarin stress. The 0.6 mM DIMBOA treatment alone also promoted the antioxidant damage capacity of alfalfa seedlings, showing an allelopathy promotion effect. Foyer et al. also suggested that increased antioxidant capacity can maintain high photosynthetic efficiency and thus promote plant growth [73]. Galano and Paradies also demonstrated that decreasing ROS, increasing antioxidant enzyme activity, and increasing antioxidant in plants can mitigate oxidative damage in plants under adversity stress [74,75]. Therefore, the results of this study showed that DIMBOA could effectively alleviate the autotoxic effect caused by coumarin and improve the resistance of alfalfa seedlings to autotoxicity.

### 5.5. Alteration of Antioxidant Enzymes Activities and Antioxidant in Alfalfa under DIMBOA and Coumarin

Allelochemicals can disrupt the redox balance in plants by stimulating the accumulation of reactive oxygen species in plants while reducing the activity of antioxidant enzymes such as SOD, POD, and CAT, ultimately leading to peroxidative damage to the plasma membrane and affecting the normal structure and function of cells [76]. In this study, there were temporal differences in the photosynthesis, root features, and physiological responses of alfalfa under different treatments. The physiological indicators of alfalfa first significantly changed at 24 h after treatment. Principal component analysis showed that coumarin treatment caused oxidative damage in alfalfa seedlings mainly by increasing H_2_O_2_, O_2_^•−^, OH^•^, and MDA while decreasing SOD, GR activity, and GSH and DHA. In contrast, when coumarin and DIMBOA were used to treat alfalfa seedlings together, they mainly maintained the redox balance by increasing CAT, POD, and APX activity and ASA, while decreasing MDA and O_2_^•−^, thereby improving the oxidative damage caused by coumarin to alfalfa seedlings. DIMBOA treatment alone increased the antioxidant capacity of alfalfa seedlings by increasing the GSSG and ASA/DHA ratio and decreasing the SP and GSH/GSSG ratio. The root system is an important part of the plant for absorbing soil water and nutrients, and the growth and development of the root system directly affects the normal growth of the whole plant [62]. In this study, the root morphological features of alfalfa showed significant changes at 72 h after treatment, and the principal component analysis showed that the correlation between coumarin treatment and various root morphological parameters was low or negative, while co-treatment of coumarin and DIMBOA on alfalfa seedlings mainly promoted alfalfa seedlings by increasing root length, mean root diameter, and root volume. DIMBOA alone could also promote the root growth and development of alfalfa seedlings by increasing the root surface area. Plants rely on photosynthesis to provide the material and energy they need for growth and development, and this is the foundation of life activities in plants [77]. In this study, photosynthetic parameters of alfalfa also changed significantly at 96 h after treatment, and the correlation between coumarin treatment and various photosynthetic indicators was low or negative, which had an inhibitory effect on the photosynthesis of alfalfa seedlings. Both coumarin and DIMBOA together enhanced the photosynthetic capacity of alfalfa seedlings, mainly by increasing Chl b and net photosynthetic rate (*Pn*). DIMBOA treatment alone also contributed to the photosynthetic capacity of alfalfa seedlings by increasing Chl (a + b) and stomatal conductance *(gs*).

## 6. Conclusions

Both coumarin and DIMBOA had concentration-dependent effects on alfalfa seedlings. The 0.0342 mM coumarin caused widespread inhibition of various indicators in alfalfa seeds and seedlings, while 0.6 mM DIMBOA promoted alfalfa seed germination and seedling growth and development. Addition of DIMBOA under coumarin stress improved the growth and development of alfalfa under coumarin stress by enhancing antioxidant capacity, improving root morphological features, and increasing photosynthetic capacity. Moreover, principal component analysis showed that the addition of DIMBOA under coumarin stress mainly increased CAT, POD, and APX activity, as well as ASA content, whereas it decreased MDA and O_2_^•−^. It also increased root length, mean root diameter, and root volume, increased Chl b and net photosynthetic rate (*Pn*) to maintain redox balance, promoted the growth and development of alfalfa seedlings, and enhanced the photosynthetic capacity of alfalfa seedlings, thus improving the growth and development of alfalfa seedlings under coumarin stress.

## Figures and Tables

**Table 1:** Coumarin and DIMBOA standard information.

Information	Coumarin	DIMBOA
Catalog No.	ZC-53633	ZTR-D459950
CAS No.	91-64-5	15893-52-4
Molecular Formula	C_9_H_6_O_2_	C_9_H_9_NO_5_
Molecular Weight	146.14	211.17
Storage	0–8 °C	−20 °C
Purity	99.97% (HPLC)	95%

## Data Availability

The data presented in this study are available on request from the corresponding author.

## Abbreviations

APX, ascorbate peroxidase; ASA, antioxidants, ascorbate; ASA/DHA, ratio of reduced to oxidized ascorbate; ASA–GSH, ascorbate–glutathione; CAT, catalase; *Ci,* intercellular CO_2_ concentration; DHA, dehydroascrobate (oxidized ascorbate); DIMBOA, 2,4-Dihydroxy-7-methoxy-1,4-benzoxazin-3-one; *E*, transpiration rate; EDTA, ethylenediamine tetraacetic acid; Gp, germination percentage; GP, germination potential; GR, glutathione reductase; *gs*, stomatal conductance; GSH, reduced glutathione; GSSH, oxidized glutathione; GSSG, oxidized glutathione; H_2_O_2_, hydrogen peroxide; MDA, malondialdehyde; NADPH, nicotinamide adenine dinucleotide phosphate; O_2_^•−^, superoxide anion free radical; OH^•^, hydroxyl free radical; PAL, phenylalanine ammonia lyase; PBS, phosphate-buffered saline; PCA, principal component analysis; *Pn,* Net photosynthetic rate; POD, peroxidase; PVP, polyvinylpyrrolidone; RI, response index; ROS, reactive oxygen species; SE, strongest promoting effect; SOD, superoxide dismutase; SS, soluble sugar; SP, soluble protein; SVI, simple vigor index; TOPSIS, technique for order of preference by similarity to ideal solution.
